# Use of telephone calls to manage glycemic control at Mexico’s northern border

**DOI:** 10.3389/fendo.2025.1420244

**Published:** 2025-04-10

**Authors:** Víctor Hugo Vázquez Martínez, Humberto Martínez Bautista, Jesús Loera Morales, Francisco Daniel Garzón Garcia, David Vélez Maldonado, Patricia Muñoz Villegas

**Affiliations:** ^1^ Instituto Mexicano del Seguro Social, Unidad de Medicina Familiar No. 33 de Reynosa, Reynosa, Tamaulipas, Mexico; ^2^ Centro de Investigación en Matemáticas, A.C. (CIMAT), Unidad Aguascalientes, Aguascalientes, Aguascalientes, Mexico

**Keywords:** glycemic control, telemedicine, telephone, type 2 diabetes mellitus, cardiovascular risk

## Abstract

**Introduction:**

Telephone calls were used for glycemic control in type 2 diabetes mellitus (T2DM) patients during the Covid-19 pandemic. The study’s objective was to determine the factors that favor glycemic control in patients with T2DM using telephone calls in the Mexico’s northern border.

**Methods:**

A retrospective cohort study was conducted with T2DM patients from the Family Medicine Unit 33 in Reynosa, Tamaulipas, from June 2021 to June 2022. The evaluation of glycemic control involved measuring glycated hemoglobin at the beginning and end of telephone follow-up. Clinical, demographic, social, and laboratory factors were analyzed using univariate and bivariate statistical methods to compare initial and final glycemic control, finally, two logistic regression models were estimated considering glycemic control as a binary variable.

**Results:**

A total of 287 participants were followed up, comprising 122 men and 165 women, where 71.78% received nine or more phone calls. Initially, 49.13% had glycemic control, but by the end of the follow-up, it increased by 7%. Females show an Odds Ratio (OR) of 0.475 (95% CI 0.269-0.838), high-density lipid levels with an OR = 0.982 (p=0.078), and 11 follow-up telephone calls with an OR = 0.403 (95% CI 0.165-0.985), which represented factors contributing to glycemic control. Poor glycemic control is more likely in individuals with a high cardiovascular risk, with an OR of 2.193 (p=0.085).

**Conclusion:**

Cell phone calls can effectively control glycemia in T2DM patients. Therefore, they can be used as a substitute for in-person medical care.

## 1 Introduction

Telemedicine is a valuable tool for delivering preventive medical care, educational services, diagnoses, or treatments when access to health services is limited by distance or other factors. In Mexico, the healthcare system was restructured during the Covid-19 pandemic to prioritize patients with Covid-19. Additionally, telemedicine programs were implemented to monitor patients with type 2 diabetes mellitus (T2DM) (1–4).

Health institutions faced a challenge providing continuous telemedicine services, particularly in Mexico’s northern border, where T2DM rates range from 12.77-18.3%. On Mexico’s northern border, the prevalence and incidence of diabetes are high due to a significant percentage of overweight and obesity (35.7% and 41.6%, respectively), inadequate eating habits, unhealthy behaviors, and a prevalence of high blood pressure reaching up to 50% (5–9).

According to recent reports, telemedicine has been successful in enhancing medication adjustment and treating comorbidities such as arterial hypertension, dyslipidemia, and heart and kidney diseases. Studies have shown that glycated hemoglobin (HbA1c%) levels can be reduced by 0.38% to 0.5% in 25% of patients. This leads to a decrease in blood pressure by 3 mmHg, improved fasting glucose levels, and a better lipid serum profile (10–17).

While efforts are being made to address T2DM on the northern border of Mexico (18–20), there is a lack of evidence on the use of telemedicine to manage diabetes in this high-risk area. Most of the research on telemedicine in diabetic patients has been conducted in Texas and California (19, 21). Given this situation, the current study aims to identify factors that lead to glycemic control in T2DM patients through telephone calls on Mexico`s northern border.

## 2 Materials and methods

A retrospective cohort study was conducted at Family Medicine Unit 33 of the Mexican Institute of Social Security in Reynosa, Tamaulipas, from June 2021 through June 2022.

### 2.1 Study population

The study involved 287 participants diagnosed with T2DM at least six months before the study and who had attended the Family Medicine Unit for six consecutive months to ensure medical follow-up. Among them, 165 were female, and 122 were men. The participants were required to satisfy specific criteria to be included in the study. These criteria included having a diagnosis of T2DM, being beneficiaries at Family Medicine Unit 33, being at least 18 years old, possessing a mobile phone, and consenting to participate in the study. The research protocol was approved by the Research Committee 2804 and Research Ethics Committee 28048 with registration number R-2023-2804-002. The participant’s data from the digital medical record was anonymized using a dissociation procedure, preventing any association with personal information or participant identification.

The exclusion criteria were patients with speech disabilities, cognitive disabilities due to dementia, and patients undergoing steroid treatment. Furthermore, participants without complete digital clinical data or full laboratory results were excluded.

### 2.2 Data collection

During the initial interview, the participants were met in person, the follow-up program was explained to them, and took their contact details to schedule monthly phone calls. If three calls were attempted in one day without a response, it would be considered an unanswered inquiry. Laboratory examinations, including HbA1c%, were performed initially and at the end of follow-up of each participant.

Information regarding the participants who met the inclusion criteria was obtained from the Family Medicine Information System via the digital medical record, including sociodemographic, clinical, and laboratory data. The data was collected through a structured questionnaire that covered various variables, including age, gender, education, occupation, marital status, weight, height, blood pressure, body mass index, HbA1c%, fasting serum glucose, total cholesterol, low-density lipoprotein cholesterol (LDL), high-density lipoprotein cholesterol (HDL) cholesterol, triglycerides, creatinine, and smoking habits.

In addition, the study evaluated cardiovascular risk using the Framingham Cardiovascular Risk Scale, which classified the risk as low, moderate, or high (22). Glycemic control was measured based on the HbA1c% level, which was considered to be less than 6.5%, as per the criteria of the American Association of Clinical Endocrinology (23). A HbA1c% level greater than 6.5% was considered poor glycemic control. All laboratory tests were performed at the beginning and the end of the follow-up.

Laboratory examinations were performed in the clinical laboratory of the Medical Unit by an automated device, Atellica^®^ Solutions from Siemens (Global Siemens Healthineers, Erlangen, Germany).

### 2.3 Statistical analysis

To initiate our research, we conducted an exploratory analysis of global and segmented data, examining the initial and final percentage measurement of HbA1c%. Categorical variables were presented as frequencies and percentages in **Tables 1**, **2**. In contrast, the median, and interquartile range (first quartile [q1] and third quartile [q3]) were used to describe the dispersion of continuous variables in **Table 3**). Special attention was given to ensure accurate data processing. The nonparametric Wilcoxon test for paired samples (24) was used to examine the change in initial and final glycemic control, which does not require normal assumption, is not influenced by outliers, and is adequate for small sample size; analogs to the Sign test for distribution compare that rely on the median. Bivariate analyses of association and correlation between initial and final glycemic control (categorized as controlled and uncontrolled) were conducted, with each one of the study factors using chi-square or Fisher’s exact tests (for frequencies less than or equal to 5 per box in the contingency table). The polychoric correlation coefficient (25) was used to determine the association and intensity of the relationship.

**Table 1:** Sociodemographic characteristics of participants.

	Total		Glycemic control by first HbA1c%				Glycemic control by last HbA1c%			
			Yes		No		Yes		No	
	n	%	n1	%	n0	%	n1	%	n0	%
Total	287	100.00%	141	49.13%	146	50.87%	163	56.79%	124	43.21%
Sex										
Male	122	42.51	66	46.81	56	38.36	80	49.08	42	33.87
Female	165	57.49	75	53.19	90	61.64	83	50.92	82	66.13
Age (years)										
< 30	25	8.71	12	8.51	13	8.90	15	9.20	10	8.06
[30, 39]	73	25.44	36	25.53	37	25.34	39	23.93	34	27.42
[40, 49]	81	28.22	40	28.37	41	28.08	45	27.61	36	29.03
[50, 59]	60	20.91	28	19.86	32	21.92	34	20.86	26	20.97
≥ 60	48	16.72	25	17.73	23	15.75	30	18.40	18	14.52
Education										
Elementary	28	9.76	15	10.64	13	8.90	19	11.66	9	7.26
Junior high school	141	49.13	63	44.68	78	53.42	76	46.63	65	52.42
Senior high school	76	26.48	40	28.37	36	24.66	44	26.99	32	25.81
Professional	42	14.63	23	16.31	19	13.01	24	14.72	18	14.52
Occupation										
Unemployed	7	2.44	3	2.13	4	2.74	4	2.45	3	2.42
Employee	217	75.61	110	78.01	107	73.29	125	76.69	92	74.19
Homemaker	43	14.98	20	14.18	23	15.75	23	14.11	20	16.13
Retired	20	6.97	8	5.67	12	8.22	11	6.75	9	7.26
Marital status										
Married	124	43.21	59	41.84	65	44.52	68	41.72	56	45.16
Divorced	43	14.98	23	16.31	20	13.70	24	14.72	19	15.32
Separated	15	5.23	7	4.96	8	5.48	9	5.52	6	4.84
Cohabitation	72	25.09	37	26.24	35	23.97	44	26.99	28	22.58
Widowed	33	11.50	15	10.64	18	12.33	18	11.04	15	12.10

**Table 2:** Clinical characteristics, comorbidities, and glycemic control of participants.

	Total		Glycemic control by first HbA1c%				Glycemic control by last HbA1c%			
			Yes		No		Yes		No	
	n	%	n1	%	n0	%	n1	%	n0	%
Total	287	100.00%	141	49.13%	146	50.87%	163	56.79%	124	43.21%
Nutrition										
Underweight	13	4.53	5	3.55	8	5.48	7	4.29	6	4.84
Normal weight	52	18.12	29	20.57	23	15.75	31	19.02	21	16.94
Overweight	108	37.63	54	38.30	54	36.99	64	39.26	44	35.48
Obesity I	84	29.27	38	26.95	46	31.51	45	27.61	39	31.45
Obesity II	29	10.10	14	9.93	15	10.27	15	9.20	14	11.29
Obesity III	1	0.35	1	0.71	.	0.00	1	0.61	.	0.00
Hypertension										
No	61	21.25	30	21.28	31	21.23	35	21.47	26	20.97
Yes	226	78.75	111	78.72	115	78.77	128	78.53	98	79.03
COVID-19										
No	99	34.49	51	36.17	48	32.88	55	33.74	44	35.48
Yes	188	65.51	90	63.83	98	67.12	108	66.26	80	64.52
Smoke										
No	197	68.64	98	69.50	99	67.81	111	68.10	86	69.35
Yes	90	31.36	43	30.50	47	32.19	52	31.90	38	30.65
Cardiovascular risk										
Low	115	40.07	56	39.72	59	40.41	61	37.42	54	43.55
Medium	84	29.27	40	28.37	44	30.14	47	28.83	37	29.84
High	88	30.66	45	31.91	43	29.45	55	33.74	33	26.61
Telemedicine										
Six calls	3	1.05	–	–	3	1.05	2	0.70	1	0.35
Seven calls	18	6.27	9	3.14	9	3.14	9	3.14	9	3.14
Eight calls	60	20.91	35	12.20	25	8.71	40	13.94	20	6.97
Nine calls	72	25.09	33	11.50	39	13.59	41	14.29	31	10.80
Ten calls	76	26.48	40	13.94	36	12.54	43	14.98	33	11.50
Eleven calls	47	16.38	19	6.62	28	9.76	21	7.32	26	9.06
Twelve calls	11	3.83	5	1.74	6	2.09	7	2.44	4	1.39

**Table 3:** Univariate analysis using median and interquartile range of demographic and clinical characteristics.

	Total (n=287)		Metabolic control by first HbA1c%				Metabolic control by last HbA1c%			
			Yes (n1 = 141)		No (n0 = 146)		Yes (n1 = 163)		No (n0 = 124)	
	Median	(q1 - q3)	Median	(q1 - q3)	Median	(q1 - q3)	Median	(q1 - q3)	Median	(q1 - q3)
First HbA1c%	6.7	(5.8 - 8)	5.8	(5.3 - 6)	8	(7.3 - 8.7)	5.9	(5.4 - 6.1)	8.2	(7.6 - 8.9)
Last HbA1c%	6.5	(5.6 - 7.5)	5.5	(5.2 - 6)	7.5	(6.8 - 8)	5.9	(5.3 - 6.2)	7.5	(7 – 8)
Age	45	(38 – 56)	45	(38 – 55)	46	(38 – 56)	46	(38 – 56)	45	(38 – 56)
BMI	28.3	(25.78 - 31.91)	28.3	(25.78 - 31.62)	28.4	(25.78 - 32.05)	28.3	(25.78 - 31.89)	28.6	(25.78 - 31.98)
Systolic Blood Pressure	138	(128 – 145)	138	(128 – 145)	136	(128 – 143)	138	(126 – 145)	136	(128 – 143)
Diastolic Blood Pressure	87	(85 – 90)	87	(85 – 90)	87	(86 – 90)	87	(85 – 90)	87	(86 – 90)
Mean Blood Pressure	103.3	(98.33 - 107.33)	103.3	(98.33 - 109.66)	103.2	(98.33 - 107.33)	103.3	(98.33 - 108.67)	103.2	(98.83 - 107.33)
Cardiovascular risk	13.7	(7.3 - 21.5)	13.7	(7.3 - 21.5)	13.7	(7.3 - 21.5)	15.9	(7.3 - 24.8)	12.7	(7.3 - 21.5)
Serum glucose	147	(129 – 176)	129	(112 – 147)	160	(140 – 190)	134	(118 – 156)	170.5	(140 – 190)
Total cholesterol	182	(150 – 305)	184	(150 – 295)	181	(146 - 309)	185	(150 - 302)	180	(145 - 306.5)
LDL cholesterol	165	(75 - 175)	165	(75 - 175)	131.5	(70 - 170)	165	(75 - 175)	165	(70 - 175)
HDL cholesterol	40	(30 - 55)	40	(30 - 50)	45	(34 - 55)	38	(30 - 50)	45	(35 - 55)
Triglycerides	180	(110 - 230)	185	(112 - 230)	177.5	(109 - 230)	180	(109 - 230)	180	(110 - 230)
Creatinine	1	(0.8 - 1)	1	(0.8 - 1)	1	(0.8 - 1)	1	0.8 - 1)	1	(0.8 - 1)
Telemedicine	9	(8 - 10)	9	(8 - 10)	9	(8 - 10)	9	(8 - 10)	10	(9 - 10)

Using univariate and bivariate analysis, as well as our clinical experience, we present two statistically valid logistic regression models that explore the factors influencing glycemic control in individuals with T2DM. The first model (M1) incorporates sex, telemedicine strategy, cardiovascular risk, education, age, and smoking. In the second model (M2), we analyze the Odds Ratio (OR) of study factors such as sex, telemedicine strategy, cardiovascular risk, education, and HDL cholesterol. Statistical significance was established for probability values (p-value) less than 0.05.

To validate the logistic regression models we explored the following analyses: 1) Specification with the Link Test approach (26), 2) Parsimony with the Akaike (AIC) and Bayesian (BIC) criteria, 3) Calibration or agreement between observed and expected with the Hosmer and Lemeshow test (27), and 4) Discrimination, correct classification of controlled and uncontrolled, with the area under the sensitivity/specificity curve known as the ROC-AUC curve, where sensitivity measures the proportion of controls correctly classified (true positives) and specificity the proportion of uncontrolled correctly classified (true negatives) by the models.

Our primary objective was to identify patients with uncontrolled diabetes and maintain high accuracy in their classification. By doing so, we can implement effective strategies to reduce the number of uncontrolled patients and improve glycemic control in patients with T2DM. All analysis was conducted using Stata 16.1 statistical software (License: 301606237830).

## 3 Results

The initial HbA1c% measurement showed that only 49.13% (141 patients) of the total population (287) had glycemic control (HbA1c% ≤ 6.5). The final measurement showed that 56.79% (163 patients) achieved control, resulting in 22 additional patients (15% of those without initial control) gaining glycemic control (**Tables 1**, **2**).

**Table 3** displays the median for centrality distribution and, q1-q3, as a dispersion estimate. These measures of centrality and dispersion remain unaffected by asymmetry or outliers. The initial HbA1c% level was 6.7% (5.8–8%), and the final HbA1c% level was 6.5% (5.6–7.5%), indicating a reduction in median and dispersion. This aligns with the intention to use telemedicine to lower the percentage of glycated hemoglobin. Similarly, the analysis of glycemic control considered continuous characteristics such as age, body mass index, blood pressure (systolic and diastolic), serum glucose, cholesterol (Total, LDL, and HDL), triglycerides, creatinine, and the telemedicine strategy. **Table 3** presents the median and dispersion (q1-q3) for these variables.

**Figure 1:**
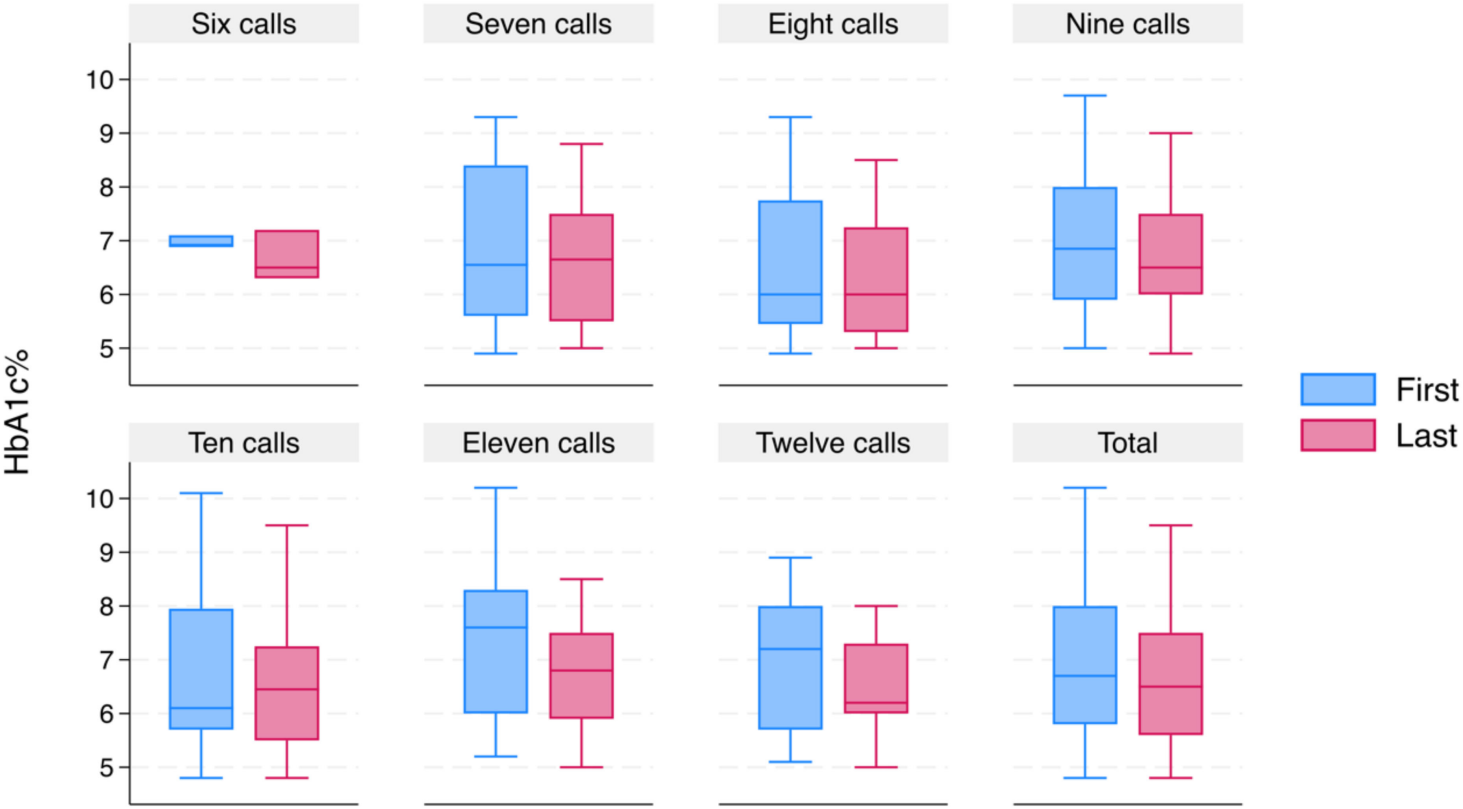
Distribution of telephone calls and change in glycated hemoglobin.

The telemedicine strategy was developed for one year and consisted of monthly follow-up through telephone calls to patients with T2DM. **Figure 1** displays the initial and final HbA1c% variations based on telemedicine intensity. In general, a reduction can be seen in its central value (median) and dispersion (variance, for q1 and q3), both of HbA1c initial and final percentage as a function of the intensity of the telemedicine strategy (number of calls). There is a noticeable decrease in HbA1c% median and variance at high-intensity levels (11 and 12 calls), which we believe is due to the implemented strategy. **Tables 1**, **2** provide the values with detailed frequencies, while **Table 3** contains information on centrality and dispersion.

According to the Wilcoxon rank paired sample test, there is a significant difference (p < 0.05) between the initial and final HbA1c% distributions. Moreover, when comparing the medians and paired samples using the sign test (28), it confirms that the decrease of 0.2% (from 6.7% to 6.5%) in median final HbA1c% compared to the initial HbA1c% is statistically significant (p < 0.05). The non-parametric tests identified a significant statistical difference (p < 0.05) in females. The results are consistent with the Chi-Square association test, which also showed an association with these levels.

**Figure 2:**
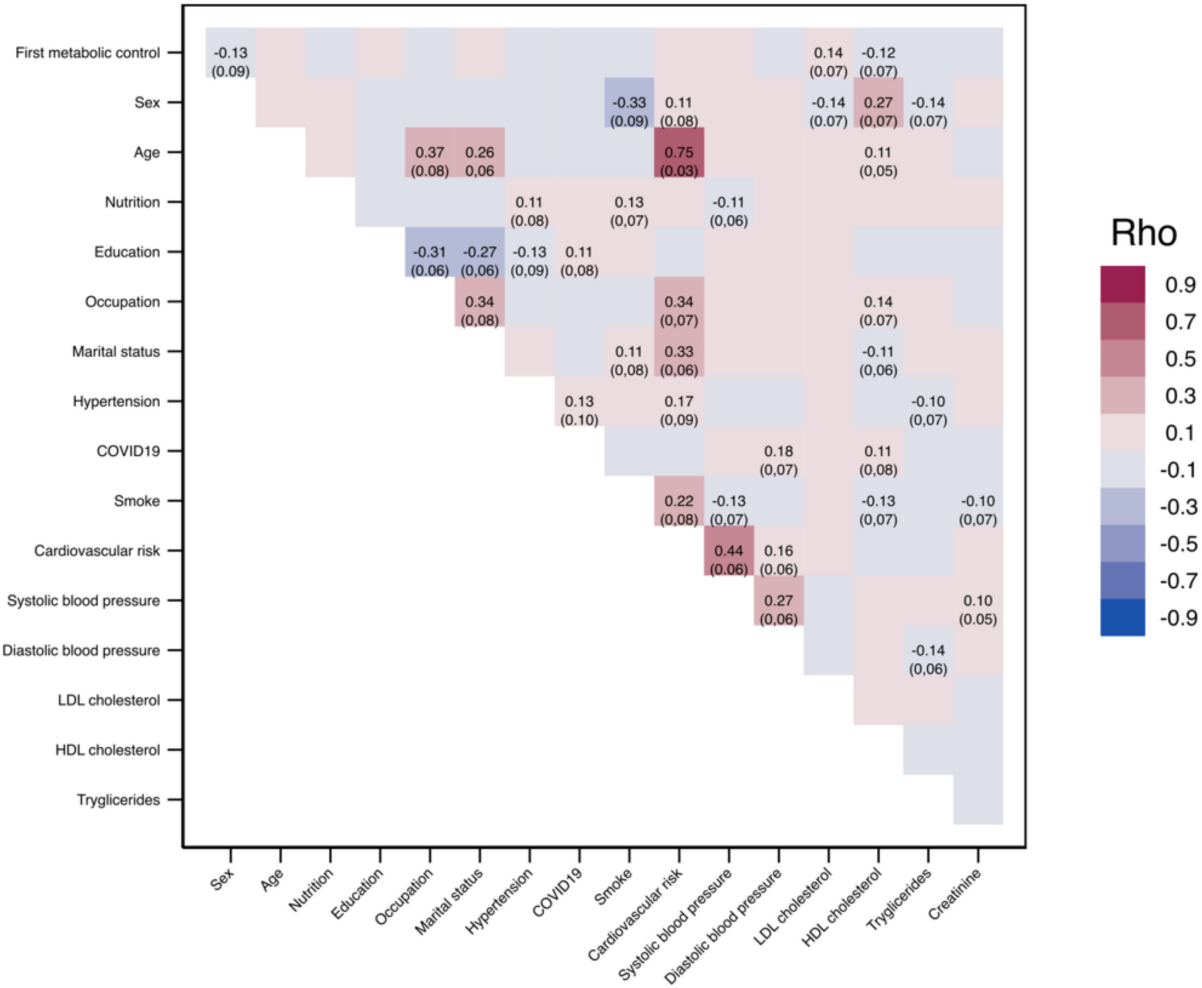
Matrix of polychoric correlations of factors on initial glycemic control.

**Figure 3:**
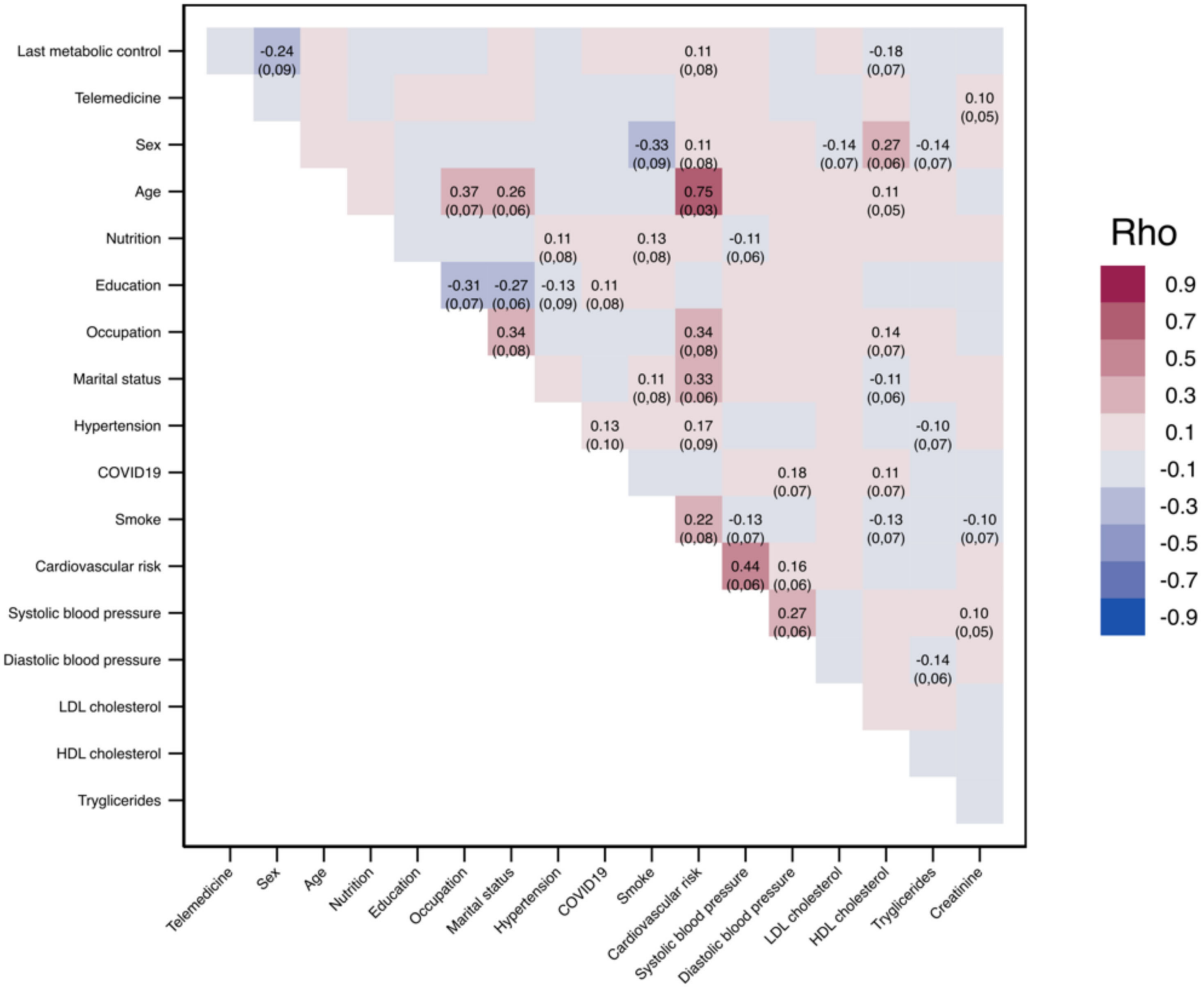
Matrix of polychoric correlations of factors on final glycemic control.

We used the polychoric correlation matrix to investigate the correlation and intensity of the factors under study on initial and final glycemic control (**Figures 2**, **3**). At the start of the study, a low negative correlation (Rho-values= -0.13 and -0.12) of initial glycemic control with factors such as sex and HDL cholesterol was observed. Additionally, we found a high positive correlation (Rho= 0.75) between age and cardiovascular risk, systolic pressure, and cardiovascular risk (Rho= 0.44). Other moderate correlations observed included sex and smoking, sex and HDL cholesterol, age and occupation, education and occupation, marital status and occupation, cardiovascular risk and smoking, and even cardiovascular risk and occupation, observing the influence of factors like sex, cardiovascular risk, and occupation on baseline metabolic control. All significant correlations are shown in **Figure 2**.

By the end of the study, the relationship between female and HDL cholesterol with the metabolic control of patients with T2DM was found to be stronger. The cardiovascular risk factor showed a low correlation but with statistical significance. These findings highlight the importance of considering these factors in studying the metabolic control of patients with T2DM. The study also confirmed the previously detected correlations, which are consistent with clinical experience (as shown in **Figure 3**). Our research hypothesis investigates if the quantity of telemedicine calls correlates with glycemic control in patients with T2DM, backed by our experience and a study conducted by Casas et al., 2023 (29).

**Figure 4:**
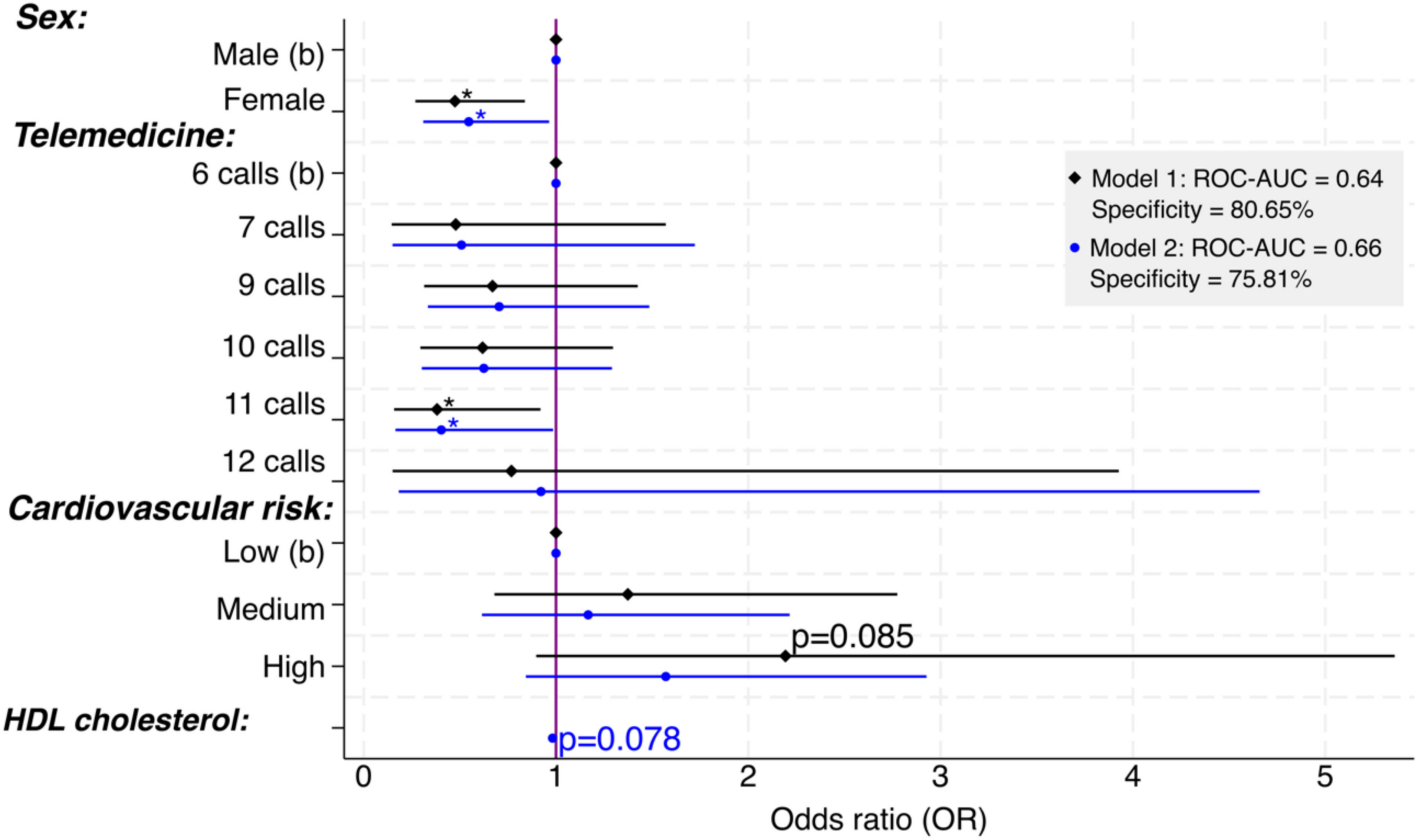
Factors that contribute to glycemic control through telephone calls. Significance: * p<.05; b = basal or reference value; 8 calls not included to avoid multicollinearity; models adjusted by age, education, and smoking were not statistically significant.

To investigate the consistency of the OR estimates in models M1 and M2, we performed the adjustment with the resampling procedure known as Bootstrap using 1000 replications, of which 973 were valid, both for M1 and M2; the results are presented in **Figure 4**, that displays the ORs for M1 and M2 simultaneously, and both models indicate consistent conclusions. Specifically, in M1, the female sex is identified as a protective factor with an OR of 0.47, similar to M2’s OR of 0.55. Women were found to offer around 50% better glycemic control protection compared to men. The telemedicine strategy was found to be a protective factor in both models, but it was only statistically significant (p < 0.05) in the intensity category -11 calls-, as compared to the low-intensity level of 6 calls. The adjustment of models M1 and M2 considers cardiovascular risk as a factor that behaves as a risk factor. There is statistical evidence of its influence in M1 for a p-value of 8.5%.

Education did not play a significant role in both models, but it helped the models fit better and has been observed to impact glycemic control in patients with T2DM. Age and smoking were added to M1 for a better fit but did not show statistical significance. Lastly, HDL cholesterol was added to M2, and with a p-value of 7.8%, it is considered significant, which aligns with clinical experience.

**Table 4** contains the indicators of specification (link test), calibration (H-L), parsimony (AIC and BIC), and discrimination (sensitivity, specificity, and ROC-AUC). Similarly, the threshold (0.623) that meets the 80% criterion for detecting patients with uncontrolled T2DM (Specificity), the target population we aim to identify to improve their care and enhance glycemic control efficiency. Despite M2 having a higher AUC-ROC, meaning better case/non-case discrimination and better parsimony (lower AIC and BIC), commonly used in health research. We emphasize the importance of using metrics with caution and alignment, especially in our research, where the primary objective is to detect uncontrolled cases. In this regard, M1 proves preferable as it correctly classifies 80.65% (100/124) of uncontrolled cases (true negatives). Likewise, the specification link test favors M1 given that the squared predictions (p_hat^2^) are less significant and, therefore, a better explanation of the predictions.

**Table 4:** Metrics to evaluate the logistic model’s performance for metabolic control in the last HbA1c%.

Model	n (Bootstrap)	Link test^1^	H-L (p-value)	AIC	BIC	cut point	Sensitivity (%)	Specificity (%)	ROC-AUC (95% CI)
M1	287 (973)	p_hat = 0.001; p_hat^2^ = 0.926	Chi^2(8)^ = 7.83 (0.45)	401.6	452.8	0.623	44.79	80.65	0.64 [0.58 – 0.71]
M2	287 (973)	p_hat = 0.000; p_hat^2^ = 0.705	Chi^2(8)^ = 2.98 (0.94)	397.2	444.7	0.623	41.72	75.81	0.66 [0.59 – 0.72]

^1^Significance predict (_hat) and square predict (_hat^2^).

H-L, Hosmer-Lemeshow goodness of fit; AIC, Akaike’s information criterion; BIC, Bayesian information criterion; ROC-AUC, the area under ROC curve; CI, confidence interval.

## 4 Discussion

### 4.1 Main findings

Patients who used the telemedicine strategy (telephone calls) had a lower percentage of glycated hemoglobin at the end of the follow-up period. The most significant benefit was observed in females and those receiving 11 telephone calls. High cardiovascular risk was a risk factor for poor glycemic control. Laboratory parameters used to measure cardiovascular risk remained constant in the initial and final measurements.

### 4.2 Comparison and contrast with other studies

Telemedicine has been used since 1970 to overcome geographical barriers and provide greater access to healthcare in developing countries and rural areas. During the COVID-19 pandemic, health systems around the world had to adapt to the new circumstances of confinement, so providing medical care for glycemic control to patients with T2DM was a real logistical challenge, especially in those areas with a high prevalence of the disease such as the Mexico’s northern border (30, 31).

Myers et al., in a randomized pilot study of telemedicine with a three-month follow-up for the control of T2DM in Hispanic and African American patients with poor glycemic control, determined that users who used the telephone showed a reduction of 2.57% in HbA1c (32). However, it is essential to note that our study design is a retrospective cohort, while that of Myers et al. is a clinical trial with a much smaller sample than the one used in this study, and they did not find a significant difference in the reduction of HbA1c between the telemedicine intervention group and standard care control.

The purpose of telemedicine is to improve accessibility to medical care in developing countries. However, it has also been used in developed countries such as Italy. Molfetta et al. in a DIAMONDS randomized clinical trial, determined that in patients with poor glycemic control, after a 6-month follow-up of self-monitoring and immediate reporting by telephone or SMS message, glucose levels had a reduction in HbA1c of -0.38% in comparison with the standard comparison group that did not use telemedicine (16). Given this, the usefulness of telephone monitoring can be observed in various scenarios, whether in developing or developed countries, demonstrating the flexibility of telephone use in the modern world. The results of Molfetta et al. on the reduction of HbA1c are similar to our results.

The use of the telephone as a method of clinical monitoring and glycemic control has not only focused on telephone calls but has also used the SMS text messaging system, as in the study carried out in Barcelona, Spain, on patients in primary services care with suboptimal glycemic control where Ortiz-Zuñiga et al. demonstrate that users under this strategy improved the level of HbA1c by -0.67% in a 4-month follow-up period (33). Telephone calls and messaging can be widely used in clinical settings with limited telephone services and suburban or rural areas with a more comprehensive digital device.

In Singapore, the clinical effectiveness of consultation by telephone compared to face-to-face by Koh et al., showed a reduction of -0.16% in glycated hemoglobin levels in patients who were seen face to face, while the decrease in HbA1c in patients seen by telephone was -0.11% (34). In China, Tan et al., conducted a 12-week prospective study in which they made 7-telephone calls to the participants. Unlike our study, these authors measured fasting plasma glucose and stratified the participants by age groups (18-40, 40-65, and over 65 years). The younger age groups saw a greater reduction in the first week. These differences were statistically significant (35). Unlike this study, our results show a greater reduction in glycated hemoglobin levels between 11 and 12 phone calls. Therefore, the positive impact of telephone calls is variable, depending on the follow-up time and the study design used.

The use of telephone calls for the management of type 2 diabetes has also been used in African countries such as Ghana, where Asante et al., in a randomized controlled pilot study comparing a telephone intervention and a traditional face-to-face intervention, demonstrated that participants who received 16 telephone calls had a -1.51% reduction in glycated hemoglobin. In contrast, the control group with face-to-face care had an increase of 0.26% (36).

One of the most representative studies in Mexico on glycemic control through telemedicine was the one developed in Tijuana, Mexico, in which Anzaldo-Campos et al., measured the impact of three intervention modalities: Dulce project, Dulce plus project mobile phone use, and traditional standard medical care, this study was able to determine that the group that had a -3.0% reduction in the HbA1c level 10-months after the study began was the Dulce project plus mobile phone use (37). The percentage reduction of HbA1% in our study was lower. However, they concur on the effectiveness of telemedicine for managing glycemic levels in patients residing along the Mexico´s northern border. This is particularly significant as our study was conducted during the pandemic, highlighting telemedicine as a valuable alternative for glycemic control in individuals with T2DM.

## 5 Conclusion

Our results contribute to telephone calls to manage glycemic control in patients with T2DM feasible in areas with a high prevalence of diabetes, like Mexico’s northern border. The effectiveness of glycemic control through telephone calls is associated with the number of phone calls received by participants; those who received more than 9 phone calls were more likely to improve the levels of HbA1c%. In addition, this may contribute to women’s health, given that women had a more significant reduction of HbA1c%. On the contrary, patients with high cardiovascular risk did not show improvement in glycemic control with this strategy (telephone calls), and there was no change (initial and final) in the laboratory parameters used to measure cardiovascular risk. Lastly, the use of telephone calls may possibly contribute to patients’ self-care and the provision of other health-care services like education, health promotion, and disease prevention in areas with high prevalence of non-communicable diseases.

### 5.1 Recommendations and weaknesses of the study

The use of information and communication technologies is changing the way medical care is provided to patients with T2DM worldwide. However, further research is needed to determine the effectiveness of alternative digital tools such as text messages, video calls, and social platforms in managing glycemic control among patients living in regions with a high prevalence of T2DM. Furthermore, it is advised to examine the access to cell phones, knowledge of their use, and the extent of the digital divide among patients who experience social marginalization, are older, and reside in remote areas. This aspect has not been addressed in the current study, which is a weakness of the report.

One of the limitations of this retrospective cohort study was the information source, which came mainly from the Family Medicine Information System and not from a source purposedly developed for the study. The categorization of the frequency of telephone calls reduced the sample between groups, which could lead to less precise information among those who received fewer phone calls than those who received 11 phone calls. Another limitation was the age of the participants; they were middle-aged individuals who commonly had access to cell phones. Therefore, a sample that included older individuals would be recommended.

## Data Availability

The raw data supporting the conclusions of this article will be made available by the authors, without undue reservation.
